# The impact of inoculation methods on bacterial aggregation and antimicrobial susceptibility testing

**DOI:** 10.1099/mic.0.001676

**Published:** 2026-03-23

**Authors:** Yu-Ming Cai, Zhi-Zhong Wang, Mark Alexander Webber, Jacob George Malone

**Affiliations:** 1 John Innes Centre, Norwich Research Park, Colney Lane, Norwich, UK; 2 Quadram Institute, Norwich Research Park, Rosalind Franklin Road, Norwich, UK; 3 School of Biomedical Engineering, Shanghai Jiao Tong University, 800 Dongchuan Rd, Shanghai 200240, PR China; 4 School of Biological Sciences, University of East Anglia, Norwich, UK

**Keywords:** aggregation, antimicrobial susceptibility testing (AST), inoculation, minimum inhibitory concentration (MIC), *Pseudomonas aeruginosa*

## Abstract

Antimicrobial susceptibility testing (AST) has been a gold standard for guiding physician treatment practices for accurate antibiotic prescription, of which the broth microdilution method is recommended and commonly used. Different inoculation methods and preparative steps of bacterial suspension for AST are applied in various settings, which are based on the assumption that planktonic broth cultures contain homogeneous single cells. However, this has been challenged by the common observation of non-surface-attached aggregates in shaking cultures. Whether different inoculation methods producing different aggregation patterns contribute to inconsistent AST results has not been systematically investigated. To address this, we evaluated how routinely used inoculation methods for *Pseudomonas aeruginosa* influence aggregation levels in liquid cultures, using 2D micrographs and novel mathematical indexes designated as the Entropic Coefficient (EC) and Dispersion Coefficient (DC). The aggregation levels of bacterial populations generated by different inoculation methods prior to AST were highly variable and were successfully differentiated by EC and DC using fluorescent or bright-field micrographs. The susceptibility of *P. aeruginosa* towards antibiotics was then measured using inocula prepared with these different methods. In line with the variations in aggregation patterns reflected by micrographs, different inoculation methods also resulted in inconsistent AST results, where, in general, directly resuspending agar colonies in broth yielded more consistent MIC results compared with other preparations. Our work indicates that different inoculation methods influence aggregation behaviours and AST results and provides rapid and inexpensive methods to quantify aggregation levels using 2D micrographs.

## Data Availability

Data will be made available on request.

## Introduction

Antibiotics are considered the foundation of modern medicine, having saved hundreds of millions of lives and greatly extending the overall life expectancy worldwide [1]. However, antibiotic-resistant bacteria have rapidly emerged since the introduction of different antibiotics. Since 2015, all known classes of antibiotics have encountered corresponding resistance genes [2,3]. Projections by the Organization for Economic Cooperation and Development (OECD) anticipate a twofold increase in resistance to last-resort antibiotics by 2035. Furthermore, the development of novel antibiotics is proceeding slowly, caused by both technical difficulties and inadequate economic investment [4]. It has been estimated that by the year 2050, antimicrobial resistance will result in ten million preventable deaths per year, costing up to United States Dollar 100 trillion lost from gross domestic product [5]. It is well recognized that misuse and overuse of antibiotics will drive the rapid development of resistance by posing selective pressures and increasing genetic mutation rates [6,7]. Despite this, antibiotics are still being overprescribed or misused worldwide [8]. Hence, appropriate selection and usage of antibiotics are essential for slowing down the development of resistance and optimizing therapeutic outcomes.

The determination of MIC for infecting micro-organisms serves as a strong support and basis for optimizing the choice and dosage of antibiotics in clinical practice, where tested micro-organisms can be classified as ‘Susceptible, standard dosing regimen’ (S), ‘Susceptible, increased exposure’ (I/SDD) and ‘Resistant’ (R) according to both the European Committee on Antimicrobial Susceptibility Testing (EUCAST) [9] and the Clinical and Laboratory Standards Institute (CLSI) [10]. In contrast, the value of minimum bactericidal concentration (MBC) in clinical settings remains controversial and limited, and it has been used primarily for evaluating the dosage required to completely eradicate infections (e.g. infective endocarditis) [11,12]. Nonetheless, MBC serves as a useful research tool to study the mode of action of novel antimicrobial agents, facilitating the definition of whether an agent is bactericidal or bacteriostatic [13]. Hence, robust and consistent MIC and MBC determination methodologies in different settings are crucial.

Culture microdilution, where a defined number of bacterial cells are inoculated into well plates containing geometrically increasing concentrations of antimicrobial agents, is one of the most commonly used methods for MIC determination [14,15]. While the guidelines for the preparation of microtitre plates containing antimicrobial agents are standardized, different methods for the preparation of the inoculum have been adopted by different users, including using resuspended agar colonies and liquid cultures [14]. Wiegand *et al*. contributed a detailed protocol for the MIC test, where bacterial suspensions in media were further categorized into overnight cultures and fresh cultures [14]. While it is known that unwanted clumps/aggregates can occur upon directly resuspending agar colonies into liquid for certain strains of certain species, there is increasing evidence that bacterial auto-aggregation frequently happens in liquid cultures [16,21]. These findings contrast with the traditional concept that pure cultures consist of homogeneous, planktonic single cells. Kragh *et al*. then discovered that different inoculation methods of *Pseudomonas aeruginosa* into Erlenmeyer flasks resulted in liquid cultures with significantly different aggregation patterns among the planktonic communities, where cultures with the most numerous and largest aggregates exhibited a much higher tolerance towards tobramycin compared with other populations [20]. Mechanical disruption, such as sonication and vortexing, has been reported to reverse the antibiotic tolerance of aggregates in liquid culture, although a more comprehensive understanding of the underlying mechanisms is required based on controversial results in different reports [20,22, 23]. It is not yet a routine practice to mechanically disrupt liquid cultures prior to antibiotic susceptibility testing. As different inoculum preparation methods have been applied for MIC and MBC determination in different laboratories or clinical settings [14,24], we hereby investigated the impact of commonly used inoculation methods on aggregation levels in broth cultures. Results showed that different culture methods result in significantly different aggregation patterns of the same *P. aeruginosa* strain grown in the same nutrient broth, which may contribute to the inconsistent MIC and MBC values. As such, evaluating the aggregation patterns of bacterial suspension samples prior to antimicrobial susceptibility testing (AST) may reduce errors in different situations. Although different methods for the quantification of 3D structured aggregates based on microscopic imaging have been in use, including the measurement of mean sizes or proportion of aggregates [19,20, 25] and plotting size distribution curves/histograms [18,26], these methods either require arbitrary thresholding for the size of aggregates or generate copious graphical data that cannot be easily compared statistically. We have previously proposed a mathematical index named Aggregation Coefficient (AC) for the measurement of bacterial aggregates [21]. However, most of the abovementioned quantification methods were based on micrographs obtained from confocal laser scanning microscopes, which are less accessible in many cases despite being better suited for imaging 3D aggregates than conventional light microscopes. To address this, we proposed here two novel mathematical indexes named Entropic Coefficient (EC) and Dispersion Coefficient (DC) to quantify these different aggregation levels rapidly based on fluorescent and bright-field 2D micrographs, respectively, which successfully reflected different aggregation levels in different samples in line with both visual judgement and size distribution histograms, therefore facilitating to overcome this technical limitation presented to clinicians and some researchers should a fast evaluation of aggregation be in demand.

## Methods

### Strains and pre-culture conditions

**Fig. 1.**
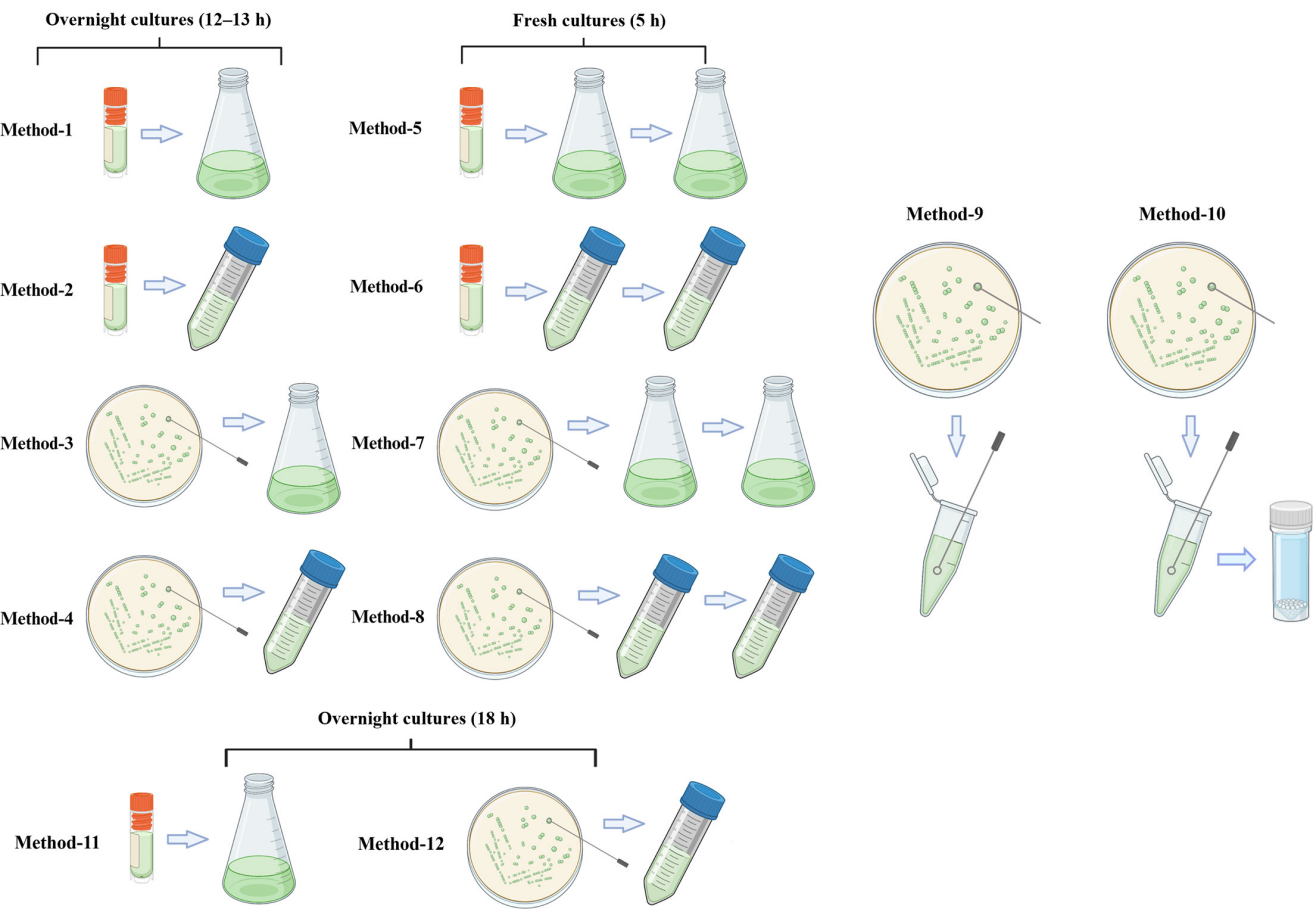
Schematic of different methods of bacterial inoculation for pre-cultures, generated by BioRender. Details of each method are described in the Methods section.

Frozen stocks of *P. aeruginosa* PAO1 or C3719 were prepared by mixing an equal volume of 50% glycerol and overnight culture in lysogeny broth (LB) grown in tubes inoculated from a single colony on LB agar (LBA). GFP-tagged PAO1 (*gfp* chromosomally inserted downstream of *glmS* with pUC18-mini-Tn7T-Gm plasmid) frozen stock was obtained in the same manner from a single colony on LBA containing 75 µg ml^−1^ gentamycin for selection, but for all growth cultures and colony suspensions using GFP-tagged PAO1, no gentamycin was used to avoid the morphological changes of cells due to antibiotic stress. To prepare for pre-cultures prior to AST, 12 different inoculation and growth methods were used in this study, as illustrated in Fig. 1. For PAO1, liquid cultures were grown in LB or Mueller–Hinton broth (MHB), while fresh overnight colonies were grown on LBA or Mueller–Hinton agar (MHA). All pre-cultures of C3719 were grown in MHB and on MHA. Method 1: overnight cultures in flasks inoculated from the frozen stock for 13 h; Method 2: overnight cultures in tubes inoculated from the frozen stock for 13 h; Method 3: overnight cultures in flasks inoculated from overnight colonies on agar plates for 13 h; Method 4: overnight cultures in tubes inoculated from overnight colonies on agar plates for 13 h; Method 5: fresh cultures in flasks inoculated from overnight cultures using Method 1; Method 6: fresh cultures in tubes inoculated from overnight cultures using Method 2; Method 7: fresh cultures in flasks inoculated from overnight cultures using Method 3; Method 8: fresh cultures in tubes inoculated from overnight cultures using Method 4; Method 9: direct resuspension of overnight colonies on agar plates; Method 10: Method 9 with 0.1 mm silica glass bead disruption (Merck BeadBug™ prefilled tubes, 2.0 ml capacity); Method 11: overnight cultures in flasks inoculated from the frozen stock for 18 h; and Method 12: overnight cultures in tubes inoculated from overnight colonies on agar plates for 18 h.

For inoculations directly from frozen stock, a loopful of frozen bacteria (10 µl loop) was used to inoculate into a flask (100 ml medium in a 250 ml glass Erlenmeyer flask) or a tube (10 ml medium in a 30 ml universal polystyrene container). For inoculations from colonies, well-separated colonies were picked each time for inoculation. All overnight cultures were grown for 12–13 h at 37 °C with 200 r.p.m. shaking, while fresh cultures were grown at the same agitation rate and temperature for 5 h by diluting overnight cultures 1 in 100 into fresh sterile medium. The volume of LB/MHB medium in each flask and tube was 100 and 10 ml, respectively. For direct resuspension of colonies in broth (Method 9), well-separated colonies were picked up by loops and resuspended in 3 ml LB/MHB medium to reach an optical density of 0.1–0.2 at 600nm wavelength, followed by vigorous pipetting for 20–30 times and 30 s of vortexing. For mechanical disruption of aggregates in resuspended colonies, 800 µl of such suspensions (OD_600nm_, 0.1–0.2) was transferred to 2 ml Merck BeadBug™ prefilled tubes, followed by 30 s of vortexing. After the vortex, tubes were left on the bench for 30 s until all beads sedimented, and samples were carefully taken from the tubes without disturbing the beads.

### Image acquisition

**Fig. 2.**
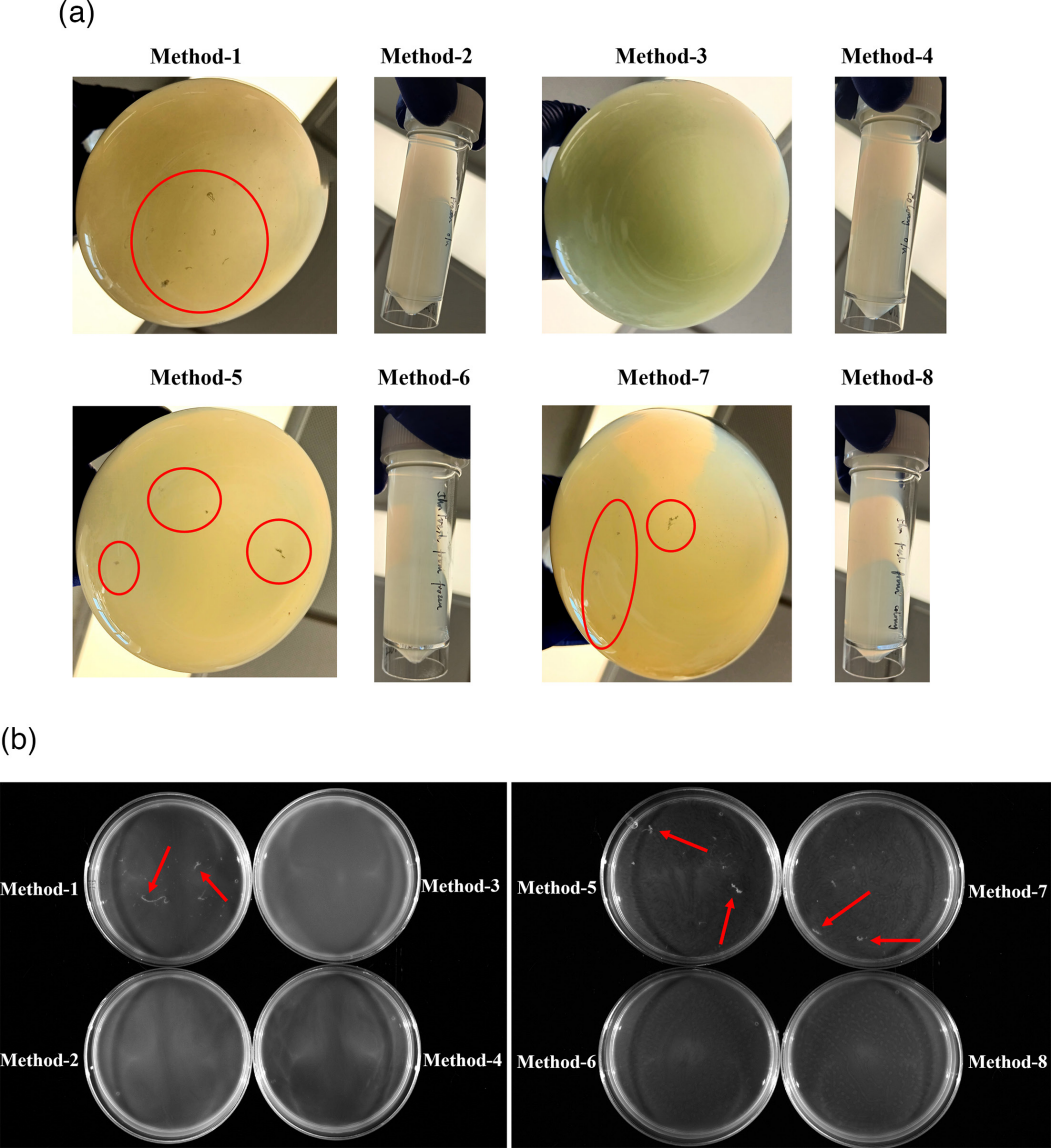
Images of visible aggregates in LB. (**a**) Bacterial cultures in their original containers grown using different methods. (**b**) Close-up observations of 5 ml bacterial cultures in Petri dishes from different inoculation methods. Visible aggregates are highlighted with red circles or arrows.

All growth cultures of GFP-tagged PAO1 (for fluorescent microscope) or C3719 (for bright-field microscope) were diluted to reach an OD_600nm_ of 0.2–0.3. A volume of 400 µl of diluted bacterial suspension was inoculated into each well in an Ibidi 24-well plate (µ-Plate 24 Well, Cat. No: 82426) with an uncoated polymer bottom. Micrographs were obtained using a 40× objective under the bright-field or GFP channel (excitation wavelength, 488 nm) on a Zeiss Axio Observer microscope. For images of aggregates in undiluted bacterial cultures in Fig. 2(b), a Bio-Rad gel imaging system (colourimetric) was used.

### Mathematical model of EC and DC, and MATLAB programming

For EC calculation, each micrograph was segmented into *N* subunits (*N*=32×32=1,024 in this study), and there are $X_{i}$ bacteria in the $i_{th}$ unit, the *Sum* of bacterial cells in each chosen micrograph is as follows:


(1.1) $$A=X_{1}+X_{2}+...+X_{i}+...+X_{N}$$

$p_{i}$ could be considered as the probability of bacterial occurrence in the $i_{th}$ subunit, and the sum of all $p_{i}$ should equal 1.


(1.2) $$p_{i}\;=\;X_{i}/A\;(i=1,2,3,...,N)$$

(1.3) $$p_{1}\;+\;p_{2}\;+\;.\;.\;.\;+\;p_{N}\;=\;1$$

Adapted from the Shannon entropy equation, the information entropy of each micrograph is defined as:


(1.4) $$H=-(p_{1}Ln\;p_{1}+p_{2}Ln\;p_{2}+...+p_{𝑁}Ln\;p_{N})$$

Based on L'Hôpital’s rule, the calculation of 𝑝Ln𝑝 when 𝑝=0 can be done as below, and 0*Ln*0 is defined as zero.


(1.5) $$\lim_{p\to 0}pLnp=\lim_{p\to 0}\frac{Lnp}{\left(1/p\right)}=\lim_{p\to 0}\frac{\left(Lnp^{\prime }\right)}{(1/p{)}^{\prime }}=\lim_{p\to 0}\frac{1/p}{-1/p^{2}}=\lim_{p\to 0}-p=0$$

When tested on a variety of micrographs, taking Log_2_$p_{i}$ or Log_10_$p_{i}$ from [4] led to the same results in equation 1.6 as below.

It is known that Shannon entropy reaches its maximum value 𝐻_max_ when 


$$p_{1}=p_{2}=\cdots =p_{N}=X_{i}/A=1/N,\text{or}$$

(1.6) $$H_{max}=(Ln\;N+Ln\;N+\cdots +Ln\;N)/N=Ln\;N$$

In contrast, Shannon entropy reaches its minimum value 𝐻_min_ when


$$p_{1}=1\text{ and }p_{2}=\ldots p_{N}=0.\;\text{Evidently}$$

(1.7) $$H_{nim}=0$$

A normalized coefficient *C* can therefore be formulated as:


(1.8) $$\begin{array}{rl} C & =(H_{max}-H)/(H_{max}-H_{min})\\ =\{Ln\;N-[-(X_{1}/A\times Ln(X_{1}/A)-(X_{2}/A\times Ln(X_{2}/A)-\cdots -X_{i}/A\times Ln(X_{i}/A)-\cdots -(X_{N}/A\times Ln(X_{N}/A)]\}/Ln\;N\\ =1-[X_{1}\times (LnA-LnX_{1})+(X_{2}\times (LnA-LnX_{2})+\cdots +X_{N}\times (LnA-LnX_{N})]/(A\times LnN)\\ =1-[A\times LnA-(X_{1}Ln\;X_{1}+X_{2}LnX_{2}+\cdots +X_{N}LnX_{N})]/(A\times LnN) \end{array}$$

Equation 1. 8 can be simplified as below and the coefficient *C* values fall within [0,1], where coefficient 𝐶=0 represents an even distribution.


(1.9) $$C=(H_{max}-H)/(H_{max}-H_{min})=1-H/LnN$$

Although coefficient *C* can distinguish different aggregation levels, the values calculated for most micrographs obtained in this study only ranged from 0 to 0.25. To enhance the contrast of numerical datasets acquired from samples with different aggregation patterns, we proposed the EC as:


(1.10) $$EC=f\times \sqrt{C}$$

where *f* is a magnifying factor to increase the resolution power (*f*=2 here).

Therefore, most EC values obtained in this study range from 0 to 1, where 0 represents a homogeneous bacterial distribution and values close to 1 represent extremely concentrated aggregation. When tested for different micrographs, the distribution histogram of EC values is more spread across the spectrum than that of coefficient *C*. Hence, the differences between the datasets generated by samples with different aggregation levels are more pronounced, which is more beneficial for statistical analysis.

Equations 1.4–1.10 were then subjected to the MATLAB programme as below:

s=0; r=log(A); rr=log(N);For i=1:N

  if x(i)§gt;0

 $s=s+\left(x\left(i\right)*\left(log\left(x\left(i\right)\right)-r\right)\right);\;\%\;where\;x\left(i\right)is\;the\;bacteria\;amount\;\in \;i^{th}\text{ pixel}$

   end

  end

*C* = 1+*s/A/rr*; *EC* = 2 *C^0.5;

For DC calculation, micrographs were first converted to binary using ImageJ (black background). The original Fourier transform decomposing a function of time into its constituent frequencies can be calculated as below:


(2.1) $$X(f)=\int_{-\infty }^{+\infty }x\left(t\right)e^{-j2\pi ft}dt$$

where time is denoted as *t* and frequency is denoted as *f*. To analyse 2D micrographs for DC calculation, images were also segregated into 32×32 units, and the 2D function representing the image was then transformed into its frequency domain representation. The output *p*(*m*, *n*) represents the amplitude and phase of each frequency component, where *m* and *n* denote discrete frequency variables. The higher frequencies represent even distribution, while the lower frequencies represent aggregation. The frequency spectrum F(*i, k*) can be calculated as below using 2D-Discrete Fourier Transform (2D-DFT):


(2.2) $$F(i,k)=2D-DFT[p(m,n)]=\;\sum_{n=0}^{N-1}\;\sum_{m=0}^{N-1}p\left(m,\;n\right)W_{N}^{im+kn}$$

(2.3) $$where\;W_{N}≜e^{-j2\pi /N}$$

The numerical output quantifying the signal distribution (bacterial aggregation level) of each micrograph can then be obtained by weighting these F(*i*, *k*) in MATLAB. The programme of calculating DC is as below:

% 2D-DFT and DC calculation

fftpic=fft2(s(:,:,2)); % p(m,n) is quantity matrix of the microscopic picture

F=abs(fftpic); % the location frequency spectrum matrix

a=F (1, 1); % the direct component of which equals the total amount

s=0; mm=N/2; m1=mm+1; % *N*=32

for j=1:m1; F(j,j)=F(j,j)/a; end

for j=2:m1; s=s+(m1-j+1)*F(j,j); end % weighting

ss=s/mm; DC=ss*ss

### Determination of MIC and MBC values

All MIC and MBC assays were performed in cation-adjusted MHB (CAMHB) (Merck). The stock solutions of different antibiotics before dilution into CAMHB were prepared as follows: 50 mg ml^−1^ tobramycin (Merck) in H_2_O; 20 mg ml^−1^ ciprofloxacin (Merck) in 0.1 M hydrochloric acid and 5 mg ml^−1^ meropenem (Thermo Fisher) in H_2_O. The tested concentrations of tobramycin for PAO1 were 0.28, 0.34, 0.43, 0.54, 0.68, 0.84, 1.05, 1.31, 1.64, 2.05, 2.56, 3.2 and 4 µg ml^−1^. The test concentrations of tobramycin, ciprofloxacin and meropenem for C3719 ranged from 0.125 to 256 µg ml^−1^ with the standard twofold series. One hundred microlitres of CAMHB broth containing different concentrations of antibiotics was mixed with 100 µl bacterial suspension containing 1×10^6^ c.f.u. ml^−1^ cells (calculated by OD_600nm_ measurement) diluted from cultures grown with different methods (Methods 1–12; Fig. 1) to reach a final inoculum of 5×10^5^ c.f.u. ml^−1^ in 96-well microtitre plates. Five microlitres of each inoculum was plated on LBA for enumeration of starting c.f.u. Plates were incubated statically at 37 °C for 18–24 h prior to absorbance measurement at OD_600nm_ on a BMG OMEGA plate reader. The lowest antibiotic concentrations that completely inhibited growth (OD_600nm_ value readings of these wells increased no more than 0.05 post-incubation compared with pre-incubation) were determined as MICs if growth could be observed in the neighbouring well containing a lower concentration of antibiotic, and if no ‘Skipped well’ was observed, following EUCAST guidelines. For MBC testing, after plate reading, bacterial suspension in each well was pipette mixed 30 times, and 20 µl was taken from each well for 10-fold serial dilution into sterile 0.9% sodium chloride. Five microlitres of each dilution was plated on LBA and incubated overnight at 37 °C for enumeration. For testing whether mechanical disruption affected the viability of PAO1 cells under our experimental settings, 5 µl of original samples prepared using Method 9 and bead-treated samples prepared using Method 10 were plated on LBA, respectively, and incubated overnight at 37 °C for enumeration.

### Statistical analysis

Three independent experiments with three technical replicates were performed for all assays. For micrograph acquisition, three random locations were imaged within each technical replicate sample. One-way ANOVA was used to compare the differences among all EC and DC values, and the differences between each two groups were compared using the post-hoc Tukey’s multiple comparisons test. Student’s t-test was used to compare the number of viable PAO1 cells with or without mechanical disruption. All statistics were performed using GraphPad Prism 10.2.3.

## Results

### Different pre-culture methods resulted in different aggregation levels of PAO1 in liquid broth

Different methods for growing *P. aeruginosa* reference strain PAO1 overnight or fresh bacterial cultures are illustrated in Fig. 1. For pre-cultures cultivated in LB prior to AST (Methods 1–9), overnight cultures were grown for 12–13 h to reach the late-exponential phase (OD_600nm_ ~ 2–2.2), except for overnight cultures in flasks inoculated from colonies, reaching OD_600nm_ ~3 after 12 h. Fresh cultures were grown for 5 h to reach the mid-exponential phase (OD_600nm_ ~ 0.6–0.8). Cultures grown for 18 h in LB, which were normally regarded as ‘overnight’, were not included here, as OD_600nm_ values of 18 h cultures in flasks inoculated from colonies were around 2.8, lower than 3 after 13 h incubation, suggesting cell death that may influence imaging and AST results. Similarly, cultures from tubes after 18 h were around 2.2, similar to those grown after 13 h, suggesting late-stationary phase or cell death.

For pre-cultures cultivated in MHB prior to AST (Methods 1–9), OD_600nm_ values of overnight cultures were lower than those grown in LB after 13 h (OD_600nm_ ~1–1.5). Therefore, the standard 18 h overnight incubation was included (Methods 11 and 12), after which pre-cultures grown in flasks reached an OD_600nm_ of 2–2.4. Fresh cultures grown in MHB reached the mid-exponential phase after 5 h (OD_600nm_ ~ 0.6–0.9).

**Fig. 3.**
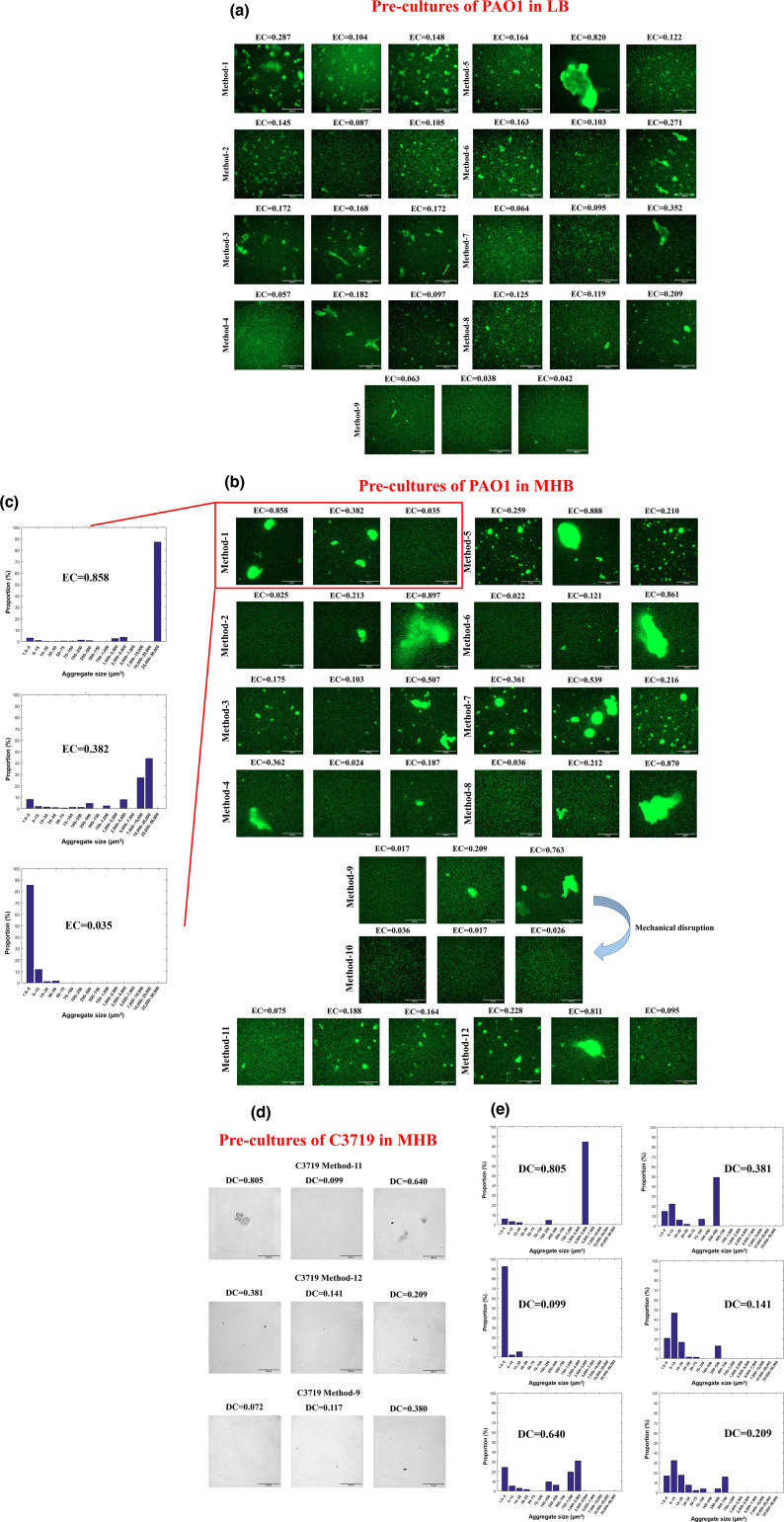
Representative micrographs of bacterial cultures from different inoculation methods diluted to OD_600nm_ 0.2–0.3. Scale bar=100 µm. (**a**) Pre-cultures of PAO1 grown in LB or on LBA using different inoculation methods. (**b**) Pre-cultures of PAO1 grown in MHB or on MHA using different inoculation methods. (**c**) Distribution of the size of PAO1 aggregates in the corresponding 3D image stacks from the same fields of view in (**b**). The interval between each slice within the stack was 0.49 µm. The biovolume (µm^3^) of single cells and aggregates was grouped into 16 categories (*x*-axis). The biovolume of all aggregates belonging to each size category was calculated, and the proportion of this biovolume of each category in the total biovolume was calculated (*y*-axis). (**d**) Pre-cultures of C3719 grown in MHB or on MHA using different inoculation methods. (**e**) Distribution of the size of C3719 aggregates in the corresponding 3D image stacks (Methods 11 and 12) from the same fields of view in (**d**). The biovolume (µm^3^) of single cells and aggregates was grouped into 16 categories. The biovolume of all aggregates belonging to each size category was calculated, and the proportion of this biovolume of each category in the total biovolume was calculated.

During bacterial growth, we observed substantial, visible clumps/aggregates in flasks after 5 or 13 h of incubation, while clumping was, in general, not observed in cultures grown in tubes by eye. Representative illustrations of visible aggregates in LB can be found in Fig. 2(a, b). We next microscopically examined the sizes of aggregates in different cultures using chromosomally GFP-tagged PAO1. For pre-cultures in LB, among the nine different methods, resuspending overnight colonies on LBA in broth yielded the most homogenous bacterial suspension, where only small aggregates could be observed occasionally (Fig. 3a; Method 9). Overnight cultures in flasks inoculated directly from −70 °C frozen stock, and the sub-cultures originating from them, contained highly variable aggregates among replicates (Fig. 3a; Methods 1 and 5). Overnight cultures in tubes directly inoculated from frozen stock (Method 2) contained much smaller aggregates compared with their counterparts in flasks, with much higher morphological consistency. By contrast, their corresponding fresh subcultures in tubes (Method 6) resulted in variable aggregates. Overnight cultures in flasks directly inoculated from colonies and their fresh sub-cultures also contained substantial aggregates (Fig. 3a; Methods 3 and 7), but the consistency among replicates was relatively high when using Method 3. Overnight cultures in tubes directly inoculated from colonies, or fresh cultures sub-inoculated from them (Fig. 3a; Methods 4 and 8), contained smaller aggregates, but the morphological variability of bacterial populations among replicates in overnight cultures was higher than in the fresh cultures.

When MHB was used, the aggregation levels of samples prepared by different methods were generally higher than those grown in LB. Highly variable aggregates were similarly found in cultures in flasks (Fig. 3b; Methods 1, 3, 5, 7). Interestingly, the aggregation patterns of bacterial suspensions cultivated in tubes displayed substantial disparity, where they, in general, either contained highly dispersed individual cells or large, concrete clumps (Fig. 3b; Methods 2, 4, 6, 8), differing from cultures in LB inoculated with the same methods. While Method 9, using fresh colonies on MHA, mainly produced highly homogeneous bacterial suspensions after vigorous pipetting and vortexing, small or substantial aggregates were also more frequently detected compared with when using colonies on LBA (Fig. 3b; Method 9). Hence, these samples were subjected to 0.1 mm silica beads and vortexed for full homogenization by mechanical disruption (Method 10). The mechanical disruption successfully dispersed aggregates, producing a highly homogeneous bacterial suspension containing almost only individual cells without damaging bacterial cell viability (Fig. S1B, available in the online Supplementary Material). The 18 h cultures in flasks from the frozen stock (Method 11) showed reduced aggregation levels compared with Method 1 incubated only for 13 h, in line with and supported by previously published data, where PAO1 aggregates dispersed upon entry into the stationary phase upon nutrient starvation [18]. However, those 18 h cultures in tubes still contained a large number of aggregates (Method 12), suggesting that stationary-phase growth is not the only factor that determines aggregate dispersal or maintenance. While the reason behind the discrepancy of aggregation patterns among cultures in tubes in MHB and LB remains to be elucidated, it can be concluded that different pre-culture methods generate highly variable aggregate patterns for PAO1 grown in two commonly used nutrient media.

We next assessed whether the same phenomenon can be observed for *P. aeruginosa* clinical isolates from patients with cystic fibrosis. C3719, the Manchester epidemic strain [27], was cultured in MHB using Methods 9, 11 and 12 as representatives. Instead of using fluorescent labelling, 2D bright-field micrographs were generated for C3719 samples to mimic what can be conveniently achieved in most clinical laboratories. As shown in Fig. 3(d), aggregates were more frequently found in overnight cultures in flasks, while samples collected from tubes or agar colony suspensions were much more homogenized, consistent with the general trending observed for PAO1 pre-cultures in LB, despite C3719 aggregates being smaller and fewer. Hence, different pre-culture methods can also lead to different aggregation patterns in bacterial cultures of clinical isolates.

### EC and DC successfully quantified and distinguished aggregation levels based on different 2D micrographs

**Fig. 4.**
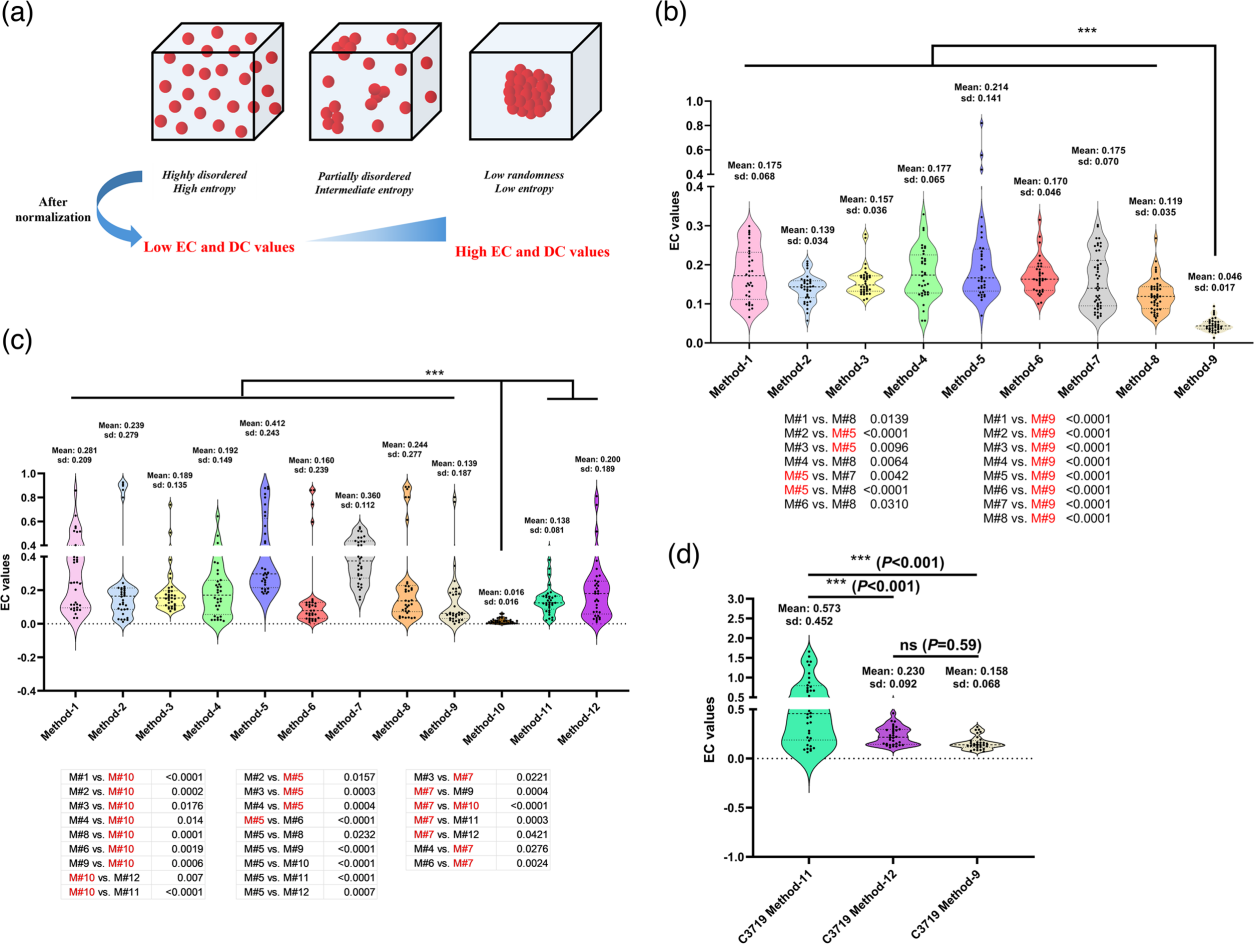
(**a**) Schematic of different bacterial aggregation patterns in liquid cultures and corresponding entropy. (**b**) and (**c**) EC values calculated from micrographs of different PAO1 bacterial culture samples inoculated with different methods in LB/LBA or in MHB/MHA, respectively. Micrographs were obtained from three independent experiments, with three technical replicates and three randomly chosen fields of view from each technical replicate. All data points are shown in the graph due to substantial variations of EC values among biological replicates and within each technical replicate. *** denotes *P*<0.01. One-way ANOVA was performed to analyse between-group and within-group variance, and post-hoc Dunnett’s multiple comparison tests were applied to compare EC values obtained between methods. In (**b**), Method 9 (direct resuspension of fresh colonies from LBA into broth) yielded the most homogeneous suspension compared with all other methods. In (**c**), Method 10 (mechanically disrupted suspension) yielded the most homogeneous suspension compared with all other methods. (**d**) DC values of C3719 pre-cultures prepared using three different methods in MHB. Micrographs were obtained in the same way as those for EC calculation mentioned above. M# denotes ‘Method-X’. Pre-culture methods highlighted in red in the statistical analysis panels resulted in significantly different aggregation patterns compared with other methods. ns denotes not significant.

As visual judgement is not quantitative, we sought to develop rapid methods to numerically assess aggregation levels using 2D micrographs. To this end, we employed two mathematical models, EC and DC, to quantify aggregation patterns in fluorescent and bright-field micrographs, respectively. The theoretical basis of EC is Shannon’s information entropy, where the disorder of a system can be measured by entropy, as described in the second law of thermodynamics. In our case, the more aggregated the bacterial community is, the higher the calculated EC value (Fig. 4a). Figs 3(a, b) and 4(b, c) demonstrated that EC successfully distinguished different aggregation levels (mean) and the heterogeneity of different samples (sd), in line with visual observations. Method 9 in LB yielded the most homogeneous bacterial suspensions, both by visual judgement (Fig. 3a) and by the lowest mean EC values with the smallest within-group variations among all methods in LB, indicating that samples were homogeneously and highly dispersed (Fig. 4b). In contrast, Method 5 in LB generated the highest variation and mean EC value (Fig. 4b), also in line with visual judgement in micrographs (Fig. 3a). On the other hand, samples from different pre-culture methods using MHB generally possessed higher mean EC values and sds compared with the corresponding methods using LB, consistent with visual judgement. When using colonies from MHA, Method 9 produced higher mean EC value with higher within-group variations compared with LB, with two outlier datasets, where substantial aggregates were found in two fields of view (Fig. 4c). As expected, after bead mechanical disruption (Method 10), the original samples prepared using Method 9 in MHB were fully homogenized, producing the lowest mean EC values with the smallest within-group variations among all methods in MHB (Fig. 4c). Method 5 in MHB also generated the highest mean EC value and high sd, similar to LB. Method 7 in MHB produced much larger aggregates than in LB (higher mean EC values), although the within-group variation was lower than most of the other methods in MHB, suggesting Method 7 in MHB generated homogeneously substantial aggregates (Fig. 4c). The occasionally detected large aggregates in Methods 2, 6 and 8 were presented as the outlier EC values in Fig. 4c.

We next determined whether our EC results corresponded to those acquired from existing aggregation assessment methods based on 3D images. Representative 3D image stacks of micrographs in the same fields of view from samples in Method 1 in MHB (Fig. 3b) were obtained, and the sizes of aggregates were calculated using the ImageJ 3D objects counter function, as previously described [28]. The histograms in Fig. 3(c) reflected the size distribution of all aggregates/individual cells within the image stack and differences in aggregation levels, in line with what was observed in Figs 3(b) and 4(c).

As fluorescent imaging is much less accessible in clinical settings, we proposed DC, using 2D-DFT, a mathematical method decomposing 2D discrete signals (e.g. digital images) into their constituent frequency components, to analyse the most frequently and conveniently obtained bright-field 2D micrographs [29]. The higher DC values represent higher aggregation levels, and lower values indicate more homogeneous samples. As shown in Fig. 4(d), calculated DC values also successfully distinguished different aggregation patterns of C3719 suspensions, as judged by the naked eye in Fig. 3(d), where cultures in flasks generated the highest mean DC values and within-group variations. It is worth noting that DC calculations were not normalized such that all values fall between 0 and 1 (i.e. highly aggregated samples can produce DC >1). Therefore, when processing micrographs containing the same aggregates, DC and EC algorithms can generate different results. When compared with existing aggregate measurement methods based on 3D micrographs, DC values also corresponded to the size distribution patterns shown in histograms (Fig. 3e). In summary, EC and DC provide easy-to-use and more accessible tools for the rapid interpretation of bacterial aggregation levels using 2D micrographs.

### Microdilution-based MIC and MBC values can be influenced by different pre-culture methods

Increased tolerance to antibiotics in the aggregated *P. aeruginosa* cells has been repeatedly reported [19,20, 30], but in most of these experiments, antibiotic challenges were posed to aggregates with a high cell density (>10^8^ c.f.u. ml^−1^). As reported earlier [17], we also observed that aggregates remain unaffected after pipetting and dilution (Fig. S1A). We therefore questioned whether different pre-cultures producing bacterial suspensions with different aggregation patterns could influence AST results. To address this, we first evaluated MIC and MBC values of tobramycin, a commonly used antibiotic for patients with cystic fibrosis (CF), against PAO1 pre-cultured using different methods (Fig. 1) in both LB and MHB prior to AST in CAMHB. Various susceptibility test results of tobramycin against PAO1 were available in multiple previous studies [31,35], which defined PAO1 as susceptible to tobramycin according to the EUCAST breakpoint. Therefore, instead of following the typical twofold dilution when preparing antibiotic solutions, we increased the resolution by using a 1.25-fold dilution, similar to that on Etest stripes, to capture the variation. As shown in Table 1, when using LB as pre-culture medium, significant variations in MIC values among different independent experiments were found when bacterial cultures were inoculated using Method 5, consistent with the high variability from both visual observations and EC values based on micrographs (Figs3a  4b). For MBC tests, all three independent experiments yielded different results when using Methods 3 and 4. Again, this inconsistency may be explained by the existence of substantial aggregates and the variability among replicates, as shown in Figs3(a)  4(b). Occasional ‘skipped wells’, mentioned in EUCAST guidelines, were observed three times from inocula using Methods 1 and 7 in LB (Table S1), in line with the heterogeneity of these cultures with high aggregation levels and high variability (Fig. 4b).

**Table 1.** MIC and MBC (µg ml^−1^) values of tobramycin against *P. aeruginosa* PAO1 with pre-cultures grown in LB or on LBA ‘Bio-1,2,3’ denotes three independent experiments, and ‘Tech-1,2,3’ denotes three technical replicates within the same experiment. Red texts highlight the different MIC/MBC values compared with other biological and/or technical replicates in the same pre-culture methods.

				Method 1	Method 2	Method 3	Method 4	Method 5	Method 6	Method 7	Method 8	Method 9
MIC	Bio-1		Tech-1	0.54	0.54	**0.54**	0.34	**0.43**	0.43	0.54	0.43	0.54
			Tech-2	0.54	0.54	**0.54**	0.34	**0.43**	0.43	0.54	0.43	0.54
			Tech-3	0.54	0.54	**0.54**	0.34	**0.43**	0.43	0.54	0.43	0.54
	Bio-2		Tech-1	0.54	**0.43**	0.43	0.34	**0.34**	0.43	0.54	**0.54**	0.54
			Tech-2	0.54	**0.43**	0.43	0.34	**0.34**	0.43	0.54	**0.54**	0.54
			Tech-3	0.54	**0.43**	0.43	0.34	**0.34**	0.43	0.54	**0.54**	0.54
	Bio-3		Tech-1	0.54	0.54	0.43	**0.43**	**0.54**	0.43	0.54	0.43	0.54
			Tech-2	0.54	0.54	0.43	**0.43**	**0.54**	0.43	0.54	0.43	0.54
			Tech-3	**0.43**	0.54	0.43	**0.43**	**0.54**	0.43	0.54	0.43	0.54
MBC	Bio-1		Tech-1	**0.68**	0.54	**0.84**	**0.43**	**0.54**	0.54	**0.68**	0.54	0.54
			Tech-2	**0.68**	0.54	**0.84**	**0.43**	**0.54**	0.54	**0.68**	0.54	0.54
			Tech-3	**0.68**	0.54	**0.84**	**0.43**	**0.54**	0.54	**0.68**	0.54	0.54
	Bio-2		Tech-1	0.54	0.54	**0.54**	**0.54**	0.68	**0.68**	0.54	0.54	0.54
			Tech-2	0.54	0.54	**0.54**	**0.54**	0.68	**0.68**	0.54	0.54	0.54
			Tech-3	0.54	0.54	**0.54**	**0.54**	0.68	**0.68**	0.54	0.54	0.54
	Bio-3		Tech-1	0.54	0.54	**0.68**	**0.84**	0.68	0.54	0.54	0.54	0.54
			Tech-2	0.54	0.54	**0.68**	**0.84**	0.68	0.54	0.54	0.54	0.54
			Tech-3	0.54	0.54	**0.68**	**0.84**	0.68	0.54	0.54	0.54	0.54

In contrast, pre-cultures in MHB generated significantly higher variations in both MIC and MBC values using all methods (Table 2) compared with LB, consistent with the higher aggregation levels demonstrated in Fig. 3(b) and generally higher mean EC values and within-group variation for each method in Fig. 4(c). Method 9 resulted in two different MIC readings summarized from three independent experiments with three technical replicates, where one independent experiment generated a lower MIC compared with the other two. Method 7 in MHB also generated more consistent results compared with other methods, with variations found in only two technical replicates, and, in total, two MIC readings were observed across all replicates, which differs from LB. Although Method 7 produced a large number of aggregates (Fig. 3b), the aggregation levels of bacterial cultures sampled across different replicates and fields of view showed less variation compared with some other methods, which was reflected by the lower sd of EC values (Fig. 4c). This may explain the higher consistency in AST results using inoculation Method 7 in MHB, though it does not necessarily mean the MIC values are more accurate. Contrary to the expectation that mechanical disruption, which fully homogenizes bacterial samples, may reduce MIC variation, Method 10 generated higher variation compared with the original samples from Method 9. It is possible that an aggressive vortexing with silica beads weakens or disrupts bacterial cell envelope structures, rendering some cells more sensitive towards antibiotics [36,38] even if cell viability was not reduced (Fig. S1B). In addition to the large variations, ‘skipped wells’ were observed 15 times using MHB among all replicates with different methods, recorded as n/a (not applicable) in Table 2, compared with only three times observed when pre-cultures were in LB, shown in Table S1. Several explanations for skipped wells were given in the EUCAST guidelines, including incorrect inoculation, contamination and heterogeneous resistance. According to our data, the chosen inoculation method may also be considered one of the factors that can be optimized for more consistent results and reduced workload. In summary, pre-cultures in MHB or from MHA led to higher inconsistency in AST results for tobramycin against PAO1 compared with LB, and different pre-culture methods contribute heavily to these variations. Resuspending PAO1 colonies from fresh overnight agar plates, followed by pipette mixture and vortexing, generally produced more homogenous bacterial suspensions and more consistent MIC readings.

**Table 2.** MIC and MBC (µg ml^−1^) values of tobramycin against *P. aeruginosa* PAO1 with pre-cultures grown in MHB or on MHA ‘Bio-1,2,3’ denotes three independent experiments, and ‘Tech-1,2,3’ denotes three technical replicates within the same experiment. Red texts highlight the different MIC/MBC values or ‘skipped wells’ (n/a) compared with other biological and/or technical replicates in the same pre-culture methods.

			M#1	M#2	M#3	M#4	M#5	M#6	M#7	M#8	M#9	M#10	M#11	M#12
	Bio-1	Tech-1	0.68	**0.84**	**n/a**	**0.68**	0.54	**0.68**	**0.54**	**n/a**	**0.68**	**0.68**	**0.84**	**0.54**
		Tech-2	**n/a**	**n/a**	**0.84**	**0.68**	0.54	**n/a**	0.68	**n/a**	**0.68**	**0.68**	0.68	**0.68**
		Tech-3	**0.84**	**n/a**	0.68	**0.68**	**n/a**	**n/a**	0.68	**0.68**	**0.68**	**0.84**	0.68	**0.54**
MIC	Bio-2	Tech-1	**n/a**	**n/a**	0.68	0.84	0.54	**0.84**	**0.54**	**0.54**	0.84	**0.54**	0.68	**0.84**
		Tech-2	0.68	0.68	0.68	0.84	**0.84**	**0.54**	0.68	**0.68**	0.84	**0.54**	0.68	**0.68**
		Tech-3	0.68	0.68	**n/a**	0.84	0.54	**0.68**	0.68	**0.84**	0.84	**0.68**	**1.05**	**n/a**
	Bio-3	Tech-1	0.68	**0.84**	0.68	0.84	0.54	**0.68**	0.68	**0.68**	0.84	**0.68**	0.68	**n/a**
		Tech-2	0.68	0.68	0.68	0.84	**0.68**	**0.54**	0.68	**0.68**	0.84	**0.54**	0.68	**n/a**
		Tech-3	**1.05**	**0.54**	0.68	**0.68**	**0.68**	**0.54**	0.68	**0.84**	0.84	**0.54**	**1.05**	**0.68**
			**M#1**	**M#2**	**M#3**	**M#4**	**M#5**	**M#6**	**M#7**	**M#8**	**M#9**	**M#10**	**M#11**	**M#12**
	Bio-1	Tech-1	**0.84**	**1.05**	**n/a**	0.68	**n/a**	0.84	**0.84**	**0.84**	0.84	**0.68**	**0.84**	**0.84**
		Tech-2	**n/a**	**n/a**	**0.84**	0.68	0.84	**n/a**	**0.68**	**n/a**	**0.68**	**n/a**	**0.84**	**0.68**
		Tech-3	**1.05**	**n/a**	**0.68**	0.68	**n/a**	**n/a**	**0.68**	**0.68**	0.84	**1.05**	**0.68**	**0.68**
MBC	Bio-2	Tech-1	**n/a**	**n/a**	**0.68**	**0.84**	0.84	0.84	**0.68**	**0.84**	0.84	**1.31**	**0.68**	**0.84**
		Tech-2	**0.68**	**1.05**	**0.84**	**1.05**	0.84	0.84	**n/a**	**0.68**	0.84	**0.84**	**0.68**	**0.68**
		Tech-3	**1.05**	**0.68**	**n/a**	**1.05**	0.84	0.84	**0.84**	**0.84**	**1.05**	**0.84**	**1.05**	**0.84**
	Bio-3	Tech-1	**0.84**	**0.84**	**1.05**	**0.84**	**0.68**	0.84	**0.68**	**0.68**	**1.05**	**0.84**	**0.84**	**0.84**
		Tech-2	**0.84**	**0.68**	**0.84**	**0.84**	0.84	**0.68**	**0.84**	**0.68**	0.84	**n/a**	**n/a**	**1.05**
		Tech-3	**1.05**	**0.68**	**1.05**	0.68	0.84	0.84	**1.05**	**n/a**	0.84	**n/a**	**1.05**	**n/a**

We then evaluated the MIC values of three antibiotics (tobramycin, ciprofloxacin and meropenem) belonging to different classes against the clinical isolate C3719, following the standard twofold dilution (Table 3). The highest intra- and inter-group consistency was observed when using meropenem, where all three methods generated the same MIC values among all replicates. For ciprofloxacin treatments, an MIC of 2 can be concluded from the overall data, although one technical replicate in the flask cultures showed an MIC of 4. In contrast, when C3719 pre-cultures were grown in flasks, two independent experiments showed that the tobramycin MIC was 4, while another one showed 2, corresponding to the larger aggregates in flask cultures. Discrepancy in tobramycin MIC values was found when using tube cultures and agar suspensions, which were 2 and 4, respectively, although the leading contributing factor does not appear to be aggregation, as both pre-culture methods generated similarly homogenized suspension with small quantities of small aggregates (Figs3d  4d). MIC measurements within one doubling dilution have been regarded as accurate in general [39]. However, as the MIC breakpoint for tobramycin against *Pseudomonas* spp. is 2 (S≤2,R§amp;gt;2) according to EUCAST breakpoint table v.15, our results suggest that different pre-culture methods may generate different MIC values, which could lead to different conclusions when defining the sensitivity of a specific clinical strain towards a specific antibiotic. Furthermore, in line with results acquired from PAO1 cultures grown in flasks, this pre-culture method can also result in higher inconsistency in AST results for clinical isolates, at least for tobramycin. Overall, our findings suggest that pre-culture methods may significantly affect AST readouts, where suspended aggregates could contribute to such variations, resulting in inaccurate readings or labour-intensive retests.

**Table 3.** MIC (µg ml^−1^) values of tobramycin, ciprofloxacin and meropenem against *P. aeruginosa* C3719 with pre-cultures grown in MHB or on MHA ‘Bio-1,2,3’ denotes three independent experiments, and ‘Tech-1,2,3’ denotes three technical replicates within the same experiment. Red texts highlight the different MIC values compared with other biological and/or technical replicates in the same pre-culture methods.

			Frozen in flask	Colony in tube	Agar colony resuspension
Tob	Bio-1	Tech-1	**2**	2	4
		Tech-2	**2**	2	4
		Tech-3	**2**	2	4
	Bio-2	Tech-1	4	2	4
		Tech-2	4	2	4
		Tech-3	4	2	4
	Bio-3	Tech-1	4	2	4
		Tech-2	4	2	4
		Tech-3	4	2	4
			**Frozen in flask**	**Colony in tube**	**Agar colony resuspension**
Cipro	Bio-1	Tech-1	2	2	2
		Tech-2	2	2	2
		Tech-3	4	2	2
	Bio-2	Tech-1	2	2	2
		Tech-2	2	2	2
		Tech-3	2	2	2
	Bio-3	Tech-1	2	2	2
		Tech-2	2	2	2
		Tech-3	2	2	2
			**Frozen in flask**	**Colony in tube**	**Agar colony resuspension**
Mero	Bio-1	Tech-1	4	4	4
		Tech-2	4	4	4
		Tech-3	4	4	4
	Bio-2	Tech-1	4	4	4
		Tech-2	4	4	4
		Tech-3	4	4	4
	Bio-3	Tech-1	4	4	4
		Tech-2	4	4	4
		Tech-3	4	4	4

## Discussion

With antimicrobial resistance (AMR) being one of the top global public health and development threats that need urgent solutions and careful management, robust methods for testing the susceptibility of clinical strains towards antibiotics are essential both for achieving optimal therapeutic outcomes for patients and slowing down global AMR. Different methods have been implemented to determine antimicrobial susceptibilities in clinical settings [40]. Although both broth microdilution and standardized disc diffusion are reference methods based on EUCAST, the accuracy and data interpretation of the disc diffusion method have been well-documented to rely heavily on operators [41,42], and many clinical laboratories have been reported to be reluctant to implement disc diffusion testing for a variety of reasons [39]. In contrast, commercialized AST kits and automated/semi-automated devices, providing ready-to-use antibiotic dilutions and software based on broth microdilution, have been developed in recent years and utilized by many clinical microbiology laboratories to facilitate swift diagnoses and reduce technical errors [39,43], where early antibiotic administration makes a substantial difference in therapeutic outcomes. Examples include MicroScan WalkAway (Beckman Coulter Inc.), Phoenix™ (Becton, Dickinson), Sensititre™ (Thermo Fisher) and Vitek2^®^ (bioMérieux) [39,43]. Despite the reduction in workflow burden and technical error using the broth microdilution method, especially in automated devices, compared with manual disc diffusion, the growth conditions of pre-cultures and their influence on the final AST results were often overlooked. Particularly, ‘skipped wells’ have been reported to result in uninterpretable MIC data/false interpretations using some of these systems [44,45], which might be contributed by the existence of substantial aggregates in pre-cultures. Although all acquired tobramycin MIC values defined PAO1 as susceptible in this study, and the inconsistent MIC values of tobramycin and ciprofloxacin against C3719 occurred only for one biological replicate and one technical replicate using flask cultures, respectively, our results, where variations of MIC readings were obtained from the same strain grown in the same batch of nutrient media with different pre-culture methods, demonstrate the heavy role of inoculation methods in the final AST result. Despite all these variations falling within one doubling dilution, which has been commonly deemed as accurate, the discrepancy of whether C3719 should be defined as susceptible or resistant to tobramycin can stem from MIC results obtained from different biological replicates using overnight cultures from frozen stocks in flasks prior to AST.

Morphological heterogeneity *per se* is far from the sole explanation for the inconsistent MIC and MBC values. Other factors, such as growth phases [46,48], heteroresistance within the population [49,50] and stochastic expression [51], can all lead to different responses to antibiotics of individual cells and endpoint MIC/MBC measurements. The growth phase was previously reported to influence the sensitivity of *Escherichia coli, Staphylococcus aureus* and *Mycobacterium smegmatis* populations towards ciprofloxacin, where this fluoroquinolone antibiotic exhibited the highest potency against exponential-phase bacterial cells and reduced activity against stationary-phase cells [47]. A similar phenomenon was observed for tobramycin (aminoglycoside) treatment against *P. aeruginosa*, where stationary-phase cells exhibited tobramycin tolerance [46]. Interestingly, the tolerance towards fluoroquinolone in *E. coli* stationary-phase cultures was not conserved in *P. aeruginosa*, as stationary-phase *P. aeruginosa* cultures showed greater susceptibility to the fluoroquinolone levofloxacin than to tobramycin and aztreonam (β-lactam) [48]. Since it is well-known that CF environments select for slow-growing clinical isolates [52,53], in our experimental design, a non-typical 12–13 h incubation period was included for PAO1 reference strain overnight cultures, to ensure that they were grown to the same late-exponential phase prior to AST as slow-growing clinical isolates, such as C3719, after standard 18 h incubation. When compared with the standard 18 h overnight cultures of PAO1 that reached stationary phase, no significant difference in the general MIC values was observed for 13 h overnight cultures in flasks (Method 1 vs. Method 11; Table 2), while 18 h overnight cultures in tubes resulted in much more frequent skipped wells compared with those grown for only 13 h (Method 12 vs. Method 4; Table 2). This may partially contribute to the conclusions that stationary-phase *P. aeruginosa* cultures are tolerant to tobramycin, as inoculation from agar colonies into Falcon tubes is one of the most used methods for pre-cultures in laboratories. Strain C3719 was grown for the standard 18 h in broth to the late-exponential stage, but according to Table 3, different pre-culture methods resulted in different tobramycin MIC values. Particularly, resuspension of agar colonies generated lower MICs compared with those grown in broth, which may be due to different growth phase from agar or in broth. Nevertheless, as using overnight cultures for AST has been a commonly seen practice in different laboratories [54], and it is impossible to control the genetic elements within a population, how the efficacy of different classes of antibiotics can be influenced by different species at distinct growth phases and the auto-aggregation patterns of a specific strain in liquid cultures should be carefully considered before AST.

In our study, in general, the most consistent AST results for two *P. aeruginosa* isolates were obtained by directly resuspending colonies into broth, followed by repeated pipetting and vortexing. However, this does not necessarily mean the same outcome would be obtained for other strains or species. When handling highly mucoid *P. aeruginosa* isolates from patients with cystic fibrosis who have chronic infections (such as NH57388A) that form substantial, concrete aggregates in liquid broth [55], we frequently failed to homogenize picked colonies in saline just by pipetting and vortexing. Therefore, the decision on whether to use fresh colonies on agar plates for MIC tests should be based on the pre-evaluation of the homogenized suspension under the microscope. When a homogenized suspension is not achievable by direct resuspension and the usage of liquid culture is essential, the different aggregation behaviours among different species using the same inoculation method should also be investigated beforehand. For instance, it was reported that while *S. aureus* cultures exhibited a similar link between inoculation methods and aggregation as seen for *P. aeruginosa*, no significant difference in aggregation was found when *E. coli* was inoculated with various methods [20]. Again, the conclusions reported here, using data from two isolates and mainly tobramycin, can only serve to illustrate the impact of the inoculation methods on AST results. Similarly, the impact of using MHB or LB as pre-culture media may have different impacts on aggregation patterns for different strains. To achieve the most consistent AST results and reduce the necessity to retest samples due to problems such as skipped wells, the choice of the specific inoculation method should be determined according to the type of antibiotic and the evaluation of aggregation level for each tested strain.

However, despite the prevalent applications of automated machines for AST in clinical laboratories based on microdilution method [39,43], evaluating the aggregation level of each pre-culture sample one by one using 3D image stacks from a microscope before AST is impossible in reality. Our novel mathematical indexes offer an easier, inexpensive and much more rapid way to assess bacterial aggregation levels in broth cultures using 2D micrographs obtained from light microscopes, especially when imaging-based AST platforms have been proposed [56,57]. Compared with our previously proposed AC [21], which requires 3D micrograph stacks, the mean acquisition and processing time of each micrograph can be reduced to less than 1 min, compared with 5–8 min when using a confocal laser scanning microscope. While even 2D imaging may be too time consuming in the face of large sample quantities, our proposed algorithm may be amenable to automation in the future via incorporation into some modern machines, such as Reshape Colony Counter (Reshape Biotech), which can image each well in 96-well plates in addition to traditional functions such as measurement of OD values. The continuously improving automated devices and upgrading software also make it highly possible to integrate such algorithms for pre-AST evaluation into models that are already widely used in hospitals, thus bridging the gap between AST in academic laboratories and clinical applications.

Although more homogenized samples tend to produce more consistent AST outputs, we showed that homogenization with mechanical disruption cannot guarantee consistent AST readings. Therefore, our results, together with the concerns about the reproducibility of AST even with automated devices in recent years [44,45, 58], point to the question of how to improve current AST methods in general, especially when it is difficult to produce a homogenized suspension for certain strains. Despite the development of a variety of alternative methods, such as MALDI-TOF MS, isothermal microcalorimetry and microdroplet analysis, which are being developed and provide more accurate measurements [59], there is still a long way to go before they can be prevalently used in all clinical settings. Traditional phenotypic ASTs are, and will still be, the golden standard in the near future due to their inherent flexibility available for bacteria, regardless of the location of infection and the nuance in interpretation that cannot be easily captured by some rapid AST methods, such as PCR detection of AMR genes [60]. On the other hand, the usage of patient-mimetic medium for AST and drug discovery has been shown to yield different results compared with those obtained from the traditional Mueller-Hinton and is considered to predict *in vivo* antibiotic susceptibility more accurately [61,64]. However, different versions of physiologically relevant media have been developed over the years (e.g. artificial sputum with various compositions [65,68]), rendering it difficult to achieve consistency across different laboratories. Hence, more in-depth understanding of the natural physiological conditions is needed before the development and determination of standardized media that match patient biopsies and body fluids. Although a detailed protocol for AST under physiologically relevant conditions has been published recently [69], the complicated procedures for preparing such media and the much stricter requirements for storage may hinder their wide usage in clinical settings. While commercialized simulated body fluids (such as different products developed by Biochemazone™) are becoming more available in recent years to address this issue, AST based on traditional media, such as MHB, still serves as the gold standard in current and foreseeable future clinical applications. As such, coupling conventional microdilution methods with pre-assessment of cell morphologies in the bacterial suspension represents a convenient way to improve the accuracy and reproducibility of the currently widely adopted AST methods worldwide.

## Supplementary material

10.1099/mic.0.001676 Uncited Supplementary Material 1.

## Abbreviations

AC: Aggregation Coefficient
AST: antimicrobial susceptibility testing
CAMHB: cation-adjusted Mueller–Hinton broth
CLSI: Clinical and Laboratory Standards Institute
DC: Dispersion Coefficient
2D-DFT: 2D-Discrete Fourier Transform
EC: Entropic Coefficient
EUCAST: European Committee on Antimicrobial Susceptibility Testing
LB: lysogeny broth
LBA: lysogeny broth agar
MBC: minimum bactericidal concentration
MHA: Mueller–Hinton agar
MHB: Mueller–Hinton broth
OECD: Organization for Economic Cooperation and Development
