# Supplementary material for: The impact of inoculation methods on bacterial aggregation and antimicrobial susceptibility testing
Source: Microbiology (Reading). 2026 Mar 23;172(3):001676. doi: 10.1099/mic.0.001676 (PMC13034079; doi:10.1099/mic.0.001676)
Supplement: Uncited Supplementary Material 1. [file mic-172-01676-s001.pdf]

Supplemental materials

Table S1 Skipped wells in MIC reading (OD_{600nm} raw data) observed in 3 independent technical replicates from Method 1 and 7 in LB.

		0	0.28μg/mL	0.34μg/mL	0.43μg/mL	0.54μg/mL	0.68μg/mL	0.84μg/mL	1.05μg/mL	1.31μg/mL
Method-1	Tech-1	1.502	1.144	0.273	0.079	0.276	0.029	0.309	0.081	0.052
	Tech-2	1.438	1.091	1.038	0.181	0.033	0.035	0.036	0.035	0.035
	Tech-3	1.437	1.08	0.757	0.069	0.029	0.034	0.034	0.035	0.037
Method-7 Bio-1	Tech-1	1.752	1.353	0.214	0.099	0.205	0.068	0.071	0.067	0.082
	Tech-2	1.788	1.35	0.179	0.17	0.038	0.031	0.03	0.032	0.03
	Tech-3	1.789	1.243	0.151	0.149	0.036	0.031	0.03	0.03	0.03
Method-7 Bio-2	Tech-1	1.804	1.481	1.101	0.209	0.064	0.069	0.073	0.258	0.085
	Tech-2	1.803	1.404	0.736	0.152	0.031	0.032	0.032	0.024	0.031
	Tech-3	1.728	1.487	0.527	0.112	0.029	0.058	0.034	0.035	0.03

Fig. S1A

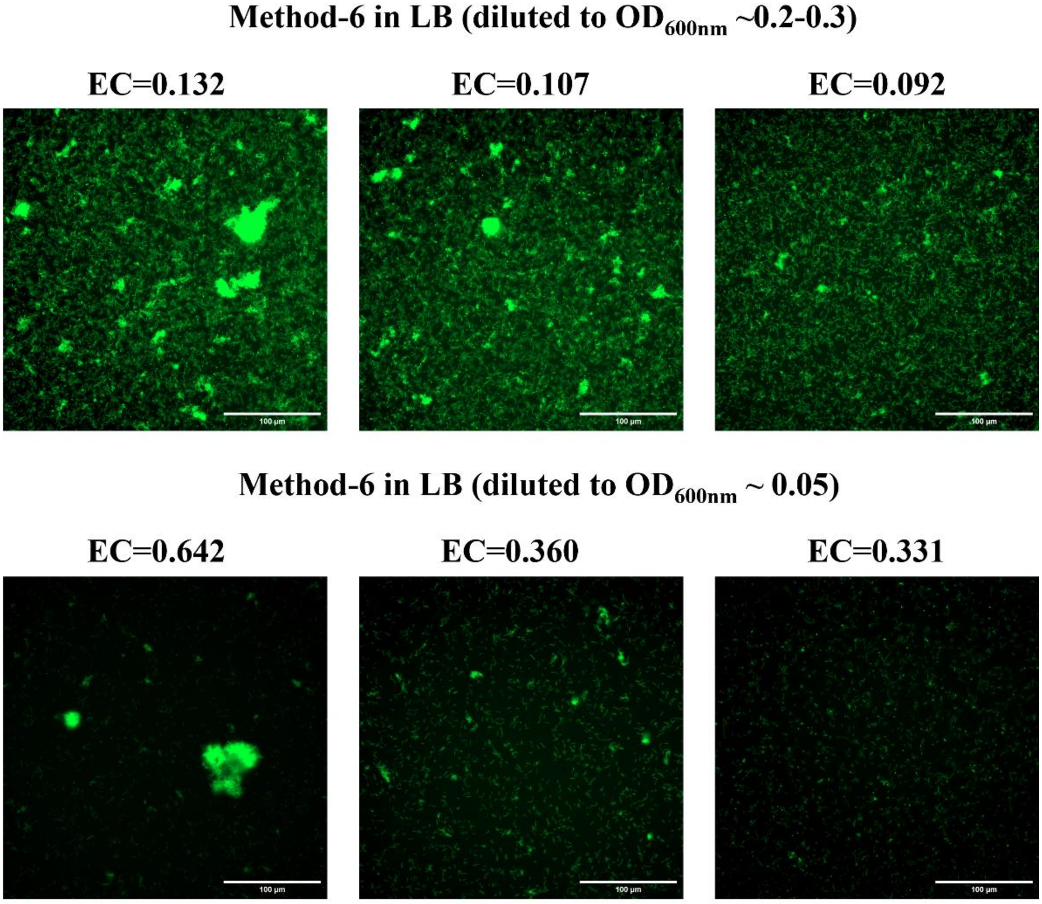

Fig. S1B

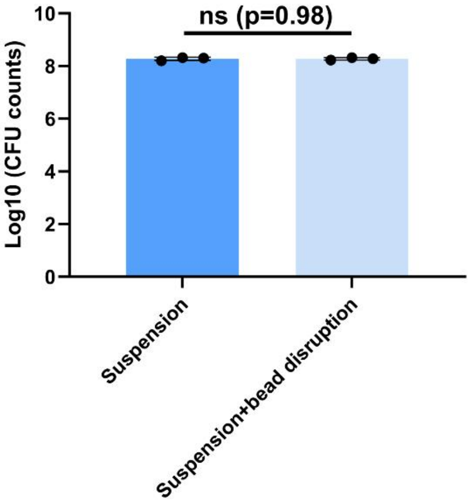

Fig. S1 (A) Representative micrographs of samples from Method-6 in LB with different dilution factors, with corresponding EC values. Upper panel: dilution factor of 2.5; Lower panel: dilution factor of 12.5. Aggregates were not disturbed after dilutions. Scale bar = 100

μm. **(B)** CFU enumeration of bacterial suspension generated from Method 9 in MHB with or without bead disruption treatment.
